# Reresection of Colorectal Liver Metastasis with Vena Cava Resection

**DOI:** 10.1155/2016/8173048

**Published:** 2016-03-20

**Authors:** Ali Tardu, Cuneyt Kayaalp, Sezai Yilmaz, Kerem Tolan, Veysel Ersan, Servet Karagul, Ismail Ertuğrul, Serdar Kirmizi

**Affiliations:** Department of Surgery, Inonu University, 44100 Malatya, Turkey

## Abstract

The best known treatment of the colorectal liver metastasis is the complete surgical excision with clean surgical margins. However, liver resections sometimes cannot appear technically feasible due to the high number of metastases in the liver, in cases of recurrent resections or invasion of the tumors to the major vascular structures or neighboring organs. Here, we presented a colorectal recurrent liver metastasis invading the retrohepatic vena cava, right adrenal gland, and right diaphragm. En masse resection of the tumor with caudate hepatectomy combined with vena cava resection and surrounding adrenal and diaphragm resections was accomplished. Caval reconstruction was done by a 5 cm in length cryopreserved vena cava homograft under isolated caval clamping. Postoperative period was uneventful and she was discharged on day 11. As a conclusion, combined liver and vena cava resection for a recurrent colorectal liver metastasis is a feasible procedure even with additional neighboring organ resections. Isolated vena cava occlusion with the preservation of the hepatic blood flow may decrease the risk of liver injury in case of previous chemotherapy for liver metastasis.

## 1. Introduction

Colorectal cancer is one of the top three most common cancers in both genders in the world [1]. In the last decades, a significant increase in the life expectancy for the colorectal cancer patients was achieved by screening programs, early diagnosis, new chemotherapeutics, and improvements in the radiation oncology and the surgical techniques [1]. Despite all these advances, poor prognosis is still a fact mainly due to the metastatic, recurrent, or locally advanced tumors. The most common organ for the metastasis of colorectal cancers is the liver and the best known treatment of the liver metastasis is a complete excision with clean surgical margins. With the help of the liver resections, the 5-year survival rates, even in cases where the surgical margin is positive or less than 1 mm, could be raised up to 25% [2]. In cases where sufficient surgical margins can be achieved (<1 mm), the 5-year survival rates could be even more than 40% [2]. However, liver resections sometimes cannot appear technically possible due to the high number of metastases in the liver, in cases of recurrent resections or invasion of the tumors to the major vascular structures or other neighboring organs. Here, we presented a liver metastasis reresection accompanying whole retrohepatic vena cava resection for a colorectal recurrent liver metastasis invading vena cava and resection of the right adrenal gland and right diaphragm. This paper was prepared by the CARE guidelines of reporting the case reports [3].

## 2. Case Report

Two years ago, a 66-year-old female (ASA score III with hypertension for 15 years) was treated by anterior resection for rectal cancer. The stage of the tumor was pT2N1M0 (IIIA) and she received adjuvant chemoradiotherapy (capecitabine oxaliplatin-50,4 Gy RT) postoperatively. After completing the adjuvant therapies, she was well without weight loss, rectal bleeding, or any change in defecation frequency. One year ago she started suffering from upper abdominal pain and the carcinoembryonic antigen (CEA) level rose to 232 ng/mL. In the colonoscopy the anastomotic line and the rest of the colonic segments were normal. Computed tomography revealed a subcapsular 5 cm in diameter metastasis between segments 6 and 7 of the liver. A PET CT (Positron Emission Tomography) for whole body screening demonstrated only one liver lesion with a SUV max value of 11.2. A segmental liver resection with clean surgical margins using Pringle maneuver and Cavitron Ultrasonic Surgical Aspirator (CUSA) was done and the patient was discharged on postoperative day four without any problem. The histopathology of the tumor was confirmed as adenocarcinoma with free surgical margins.

**Figure 1:**
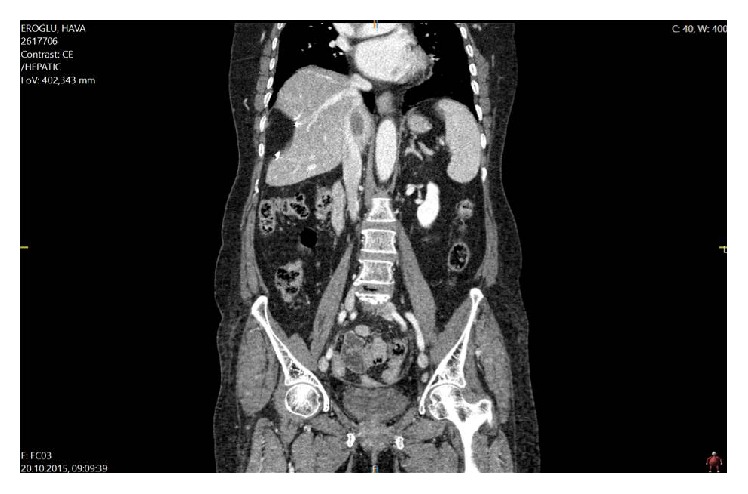
Sagittal section of a mass lesion at the caudate lobe compressing IVC. First resection site is also seen.

**Figure 2:**
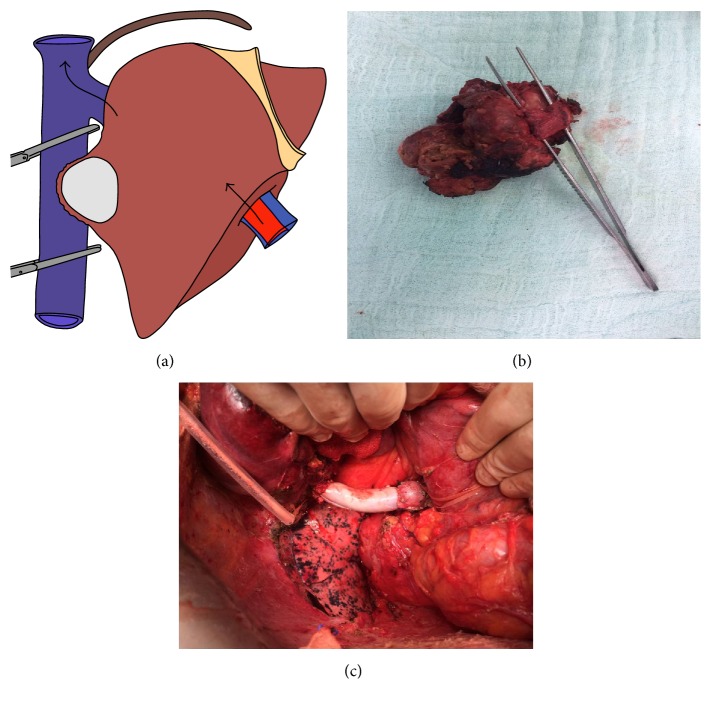
(a) A schematic drawing of isolated vena cava clamping. (b) Completely resected specimen (invaded IVC segment that was resected is included). (c) Reconstructed IVC: right diaphragm resection was performed and right lung can be seen.

**Figure 3:**
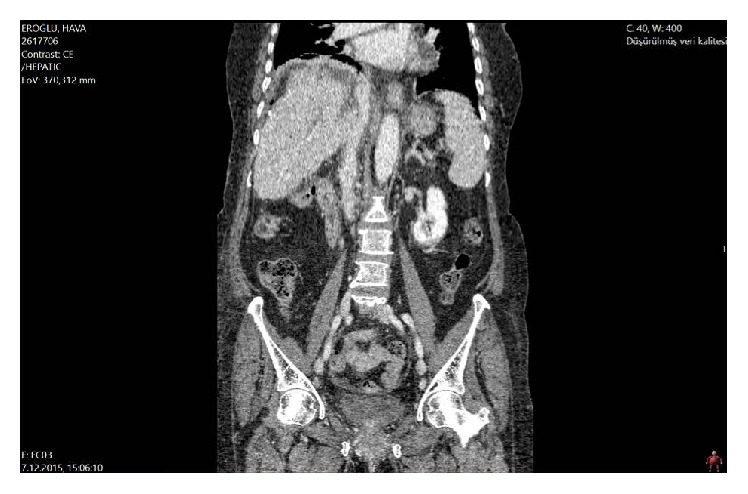
The postoperative sagittal section of the abdominal CT. IVC is seen as patent.

Thirteen months after the liver surgery, her CEA was measured as 9.86 ng/mL in the routine follow-up and CT demonstrated a 5 × 3 cm metastasis at the caudate lobe of the liver compressing on the retrohepatic vena cava (Figure 1). During surgery, the adhesions were released and the liver was fully mobilized. The caudate lobe mass was invading the right adrenal gland, the right diaphragm, and circumferentially (<50%) the retrohepatic vena cava. At first, the liver parenchyma division of the caudate lobe was done under total hepatic vascular exclusion (15 minutes, with crash clamp technique). Doing the hepatic transection at first enabled a clear determination of the tumor in the center of a mass including the vena cava, adrenal gland, and the diaphragm. An isolated caval clamping (22 minutes) by preservation of the hepatic blood flow was preferred (Figure 2(a)). No venovenous bypass was used. En bloc resection of vena cava with the liver tumor, right adrenal gland, and right partial diaphragm was performed (Figure 2(b)). Caval reconstruction was done by using a 5 cm in length cryopreserved vena cava homograft (Figure 2(c)). A tube thoracostomy was placed into the right thorax and the diaphragmatic defect was repaired with a polypropylene mesh. Total surgery time was 560 minutes. Total blood loss was around 550 mL and two units of blood transfusions were made during surgery. In the postoperative period a low molecular weighted heparin was started. In the first postoperative day, a 10 mL bile stained drainage occurred but ceased itself in the second day. The liver enzymes were elevated in the early postoperative period but they gradually decreased. The thorax tube was removed on day six, a control CT on day nine demonstrated the patent vena cava (Figure 3), and she was discharged on day 11 uneventfully. Her last CEA value was 3.58 ng/mL and she was referred to the medical oncology clinic for adjuvant therapy again. Histopathological examination confirmed the adenocarcinoma invading the vena cava, adrenal gland, and the diaphragm. The resection margins of the specimen were free of tumor. She had no problems after two months' follow-up.

## 3. Discussion

The advancements in the surgical techniques and anesthesiology make it possible to perform complex liver resections that are involving the major vascular structures. Today the cancer invading the vena cava can be no longer accepted as a contraindication in the liver metastasis surgery. In one of the most comprehensive series including concomitant liver and vena cava resections, the mortality, morbidity, and 5-year survival rates were reported as 11%, 40%, and 37.7%, respectively [4]. A recent systematic review including 204 patients who had concomitant vena cava and liver resections for any reason demonstrated that the vena cava reconstruction after resection was made with a primary closure in 40% and in the 12% a patch was used for the defect [5]. In the remaining 98 cases, a circumferential vena cava resection was required and they were repaired by synthetic grafts but only in one case a homograft was used for caval replacement just like our case. We use homografts from deceased donors frequently in our daily surgical practice, as a high volume liver transplant center (more than 200 cases per year). Here we again preferred a cryopreserved vena cava homograft due to our previous experiences with sufficient vascular patency rates [6, 7].

As far as we know there were only three reported cases that had a liver reresection and a concomitant vena cava resection [5, 8, 9]. The difficulties of the repeated liver resections were all mentioned in those reports and the risks were augmented with the vena cava resections. In one case from UK, after a caudate lobe resection with caval resection, the vena cava was replaced with a 20 mm in diameter synthetic graft [5]. During the surgery, total 60-minute hepatic inflow occlusion with intervals and a 20-minute total hepatic vascular occlusion had been required [5]. Another patient from US with an intrahepatic cholangiocarcinoma and extensive caval involvement of a recurrent tumor was reported [8]. This case was treated by chemotherapy at first and combined liver and cava resection; however, the details of this case were not available. The last case was from Italy and it was treated by liver transection at first with a 12-minute hepatic inflow occlusion and a total hepatic vascular exclusion for 25 minutes [9]. Following the caval resection, reconstruction was established with a 20 mm synthetic graft. Different from these cases, in our case we performed a cava replacement after an isolated VCI clamping without a venovenous bypass (Figure 2). Our aim in isolated caval clamping was to reduce the probability of the liver injury of the patient who had a previous chemotherapy. Even though the hepatic inflow occlusion time was only 15 minutes, postoperative liver function tests deteriorated but fortunately improved in a short period of time. Again differing from the other reported cases, we performed additional right adrenalectomy and diaphragm resection with the accompanying hepatectomy and caval resection. Despite the technical feasibility of the complex procedure, the follow-up period of our case was very short for the oncological result.

As a conclusion, combined liver and vena resection for a recurrent colorectal liver metastasis is a feasible procedure even with additional adrenal gland and diaphragm resection. Isolated vena cava occlusion with the preservation of the hepatic blood flow may decrease the risk of liver injury.
